# Evaluation of Drinking Water Disinfectant Byproducts Compliance Data as an Indirect Measure for Short-Term Exposure in Humans

**DOI:** 10.3390/ijerph14050548

**Published:** 2017-05-20

**Authors:** Shahid Parvez, Kali Frost, Madhura Sundararajan

**Affiliations:** 1 Department of Environmental Health Science, Indiana University Fairbanks School of Public Health, 1050 Wishard Boulevard, Indianapolis, IN 46202, USA; kdfrost@iu.edu; 2 Department of Epidemiology, Indiana University Fairbanks School of Public Health, 1050 Wishard Boulevard, Indianapolis, IN 46202, USA; madsunda@iu.edu

**Keywords:** disinfection byproducts, drinking water, Trihalomethanes, Haloacetic acids, temporal variability, exposure assessment, locational running annual average, birth outcomes

## Abstract

In the absence of shorter term disinfectant byproducts (DBPs) data on regulated Trihalomethanes (THMs) and Haloacetic acids (HAAs), epidemiologists and risk assessors have used long-term annual compliance (LRAA) or quarterly (QA) data to evaluate the association between DBP exposure and adverse birth outcomes, which resulted in inconclusive findings. Therefore, we evaluated the reliability of using long-term LRAA and QA data as an indirect measure for short-term exposure. Short-term residential tap water samples were collected in peak DBP months (May–August) in a community water system with five separate treatment stations and were sourced from surface or groundwater. Samples were analyzed for THMs and HAAs per the EPA (U.S. Environmental Protection Agency) standard methods (524.2 and 552.2). The measured levels of total THMs and HAAs were compared temporally and spatially with LRAA and QA data, which showed significant differences (*p* < 0.05). Most samples from surface water stations showed higher levels than LRAA or QA. Significant numbers of samples in surface water stations exceeded regulatory permissible limits: 27% had excessive THMs and 35% had excessive HAAs. Trichloromethane, trichloroacetic acid, and dichloroacetic acid were the major drivers of variability. This study suggests that LRAA and QA data are not good proxies of short-term exposure. Further investigation is needed to determine if other drinking water systems show consistent findings for improved regulation.

## 1. Introduction

Although the use of disinfectants (e.g., chlorine or chloramine) in drinking water to control microbial pathogens (e.g., *E. coli* and *Cryptosporidium*) is widely considered one of the greatest advances in public health in the 20th century [1], these disinfectants react with natural organic matter and halide salts in the treatment process and form undesirable disinfectant byproducts (DBPs). At present, nearly 600 DBPs have been identified and nearly 80 DBPs have been measured in drinking water [2,3,4]. Trihalomethanes (THMs) were the first group of DBPs detected in finished drinking water and have been regulated since 1979 due to their association with elevated chronic cancer risk [5]. In 1998, haloacetic acids (HAAs) were regulated as the second group of DBPs due to their frequent occurrence in the disinfected water supply [6]. The regulated individual THMs include chloroform (TCM), bromodichloromethane (BDCM), dibromochloromethane (DBCM), and bromoform (TBM); individual regulated HAAs include monochloroacetic acid (MCAA), dichloroacetic acid (DCAA), trichloroacetic acid (TCAA), monobromoacetic acid (MBAA), and dibromoacetic acid (DBAA). These regulations were written based on the association between long-term chronic DBP exposure via drinking water consumption and bladder, colorectal, and liver cancer risk [7,8,9,10]. To meet the compliance standards for regulated THMs and HAAs, community water suppliers are advised to adhere to a recommended sampling protocol to measure chronic long-term exposure.

As per the Stage 2 DBP Rule set by the EPA (U.S. Environmental Protection Agency) (2002), each community water supplier needs to calculate the locational running annual average (LRAA) at locations throughout the distribution system which have been identified as having high concentrations of THMs and HAAs. These higher concentration locations are identified through an Initial Distribution System Evaluation, which is required for larger water systems (serving >10,000 people) and uses historic data or models for identification of the locations. The LRAA compliance data on THMs and HAAs are derived using the QA of all samples taken from each monitoring location in the distribution system. QA data for the current quarter is combined with the three preceding quarterly running averages to obtain the LRAA for the system across four quarters. Although this represents an improvement over the system-wide averaged data (as per DBP Rule-1), the compliance data remains sparse due to quarterly, or in some cases, annual sampling requirements (for water suppliers servicing <10,000 people) [11]. It is suspected that the existing LRAA compliance data do not account for the seasonal temperature (summer vs. winter) and spatial fluctuations, source water characteristics (surface, ground, or mixed), and disinfection method (chlorination, chloramination, ozonation) [12,13,14], which influence THMs and HAAs formation. These temporal and spatial variabilities reveal exposures as short as a few weeks and as long as a few months and their high exposure levels are suspected to affect a developing fetus. There are several epidemiologic studies that suggest that prenatal DBP exposure is associated with adverse birth outcomes (Table 1). Several of these epidemiologic studies reported an association between exposure to THMs and/or HAAs and an increased risk of birth anomalies, adverse fetal growth, and small gestational duration [15,16,17,18,19,20,21,22]. Most of these studies have observed these associations at much lower levels than the EPA permissible limits of regulated THMs and HAAs [17,18,22,23]. However, without good quality short-term acute exposure data on THMs and HAAs, the relationship cannot be confirmed. The only existing study that used more frequently collected drinking water data showed a correlation with preterm delivery, a suggestive finding [24]. A few studies have used survey techniques to capture inter-individual behavioral differences and drinking water intake, but most do not go beyond reported compliance data on THMs and HAAs levels.

There are two challenges with compliance data. First, the frequency of collection: annual or quarterly, may not capture short-term but high levels of THMs and HAAs present in participants’ drinking water. Second, most community water suppliers report the total (aggregated) concentration levels of regulated THMs and HAAs. The existing compliance requires aggregated levels of THMs and HAAs, rather than exposure levels of individual THMs and HAAs. Individual levels of these DBPs may show substantial variability in exposure levels in different environmental conditions. The handful of studies that use exposure data on individual regulated DBPs indicate improved associations with adverse birth outcomes among brominated DBPs [13,17,18]. Brominated THMs, including TBM and DBCM, and brominated HAAs, including DBAAs, are considered more genotoxic and mutagenic than chloro-derivatives of THMs and HAAs, and aggregated data mask these distinctions [3,32,33,34,35,36].

Therefore, we conducted a study with two aims. First, to evaluate whether LRAA compliance and QA data adequately represent short-term exposure levels of total THMs and HAAs in the peak DBP season (i.e., May–August, when high temperature and rainfall in the study area lead to high DBP formation). Second, to determine the exposure variability in individual DBPs that exposure assessment should account for.

## 2. Materials and Methods

We designed a residential tap water monitoring plan to collect residential tap water samples from multiple locations in a large water distribution system and compared the levels of THMs and HAAs with QA and LRAA compliance data. We also evaluated the short-term exposure variability of individual regulated DBPs to determine their influence on aggregated THM and HAA levels. We preferred residential tap water samples over water samples collected from a treatment facility because they account for the spatially formatted excess THMs and HAAs in the distribution system due to excess contact time of natural organic matter with residual chlorine.

### 2.1. Description of the Community Water System

A large community drinking water distribution system that serves nearly 1 million people in a major midwestern city in the United States was chosen for this study. We included five water treatment stations (A through E) in the distribution system, wherein each station gets water from a separate source water body. The treatment stations that receive raw water from surface water bodies are A, B, C, and D; E receives groundwater. All treatment stations use chloramine as the primary disinfection method. Table 2 provides historic data from 2011–2015 depicting the main physical and chemical characteristics for each source water body (raw water) and treatment stations (finished water).

**Figure 1:**
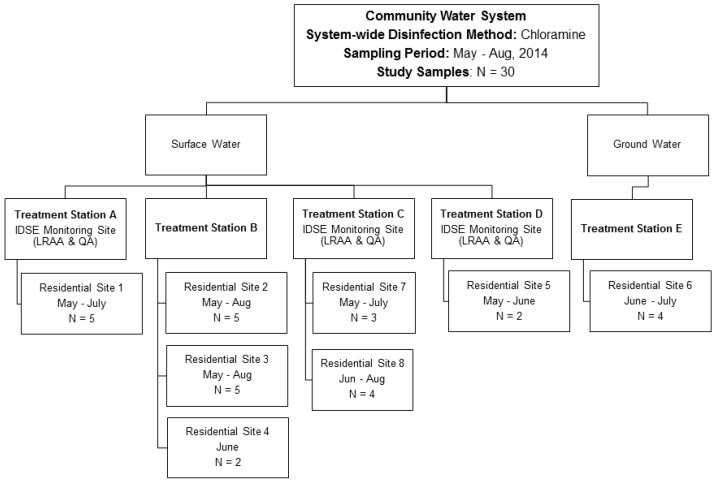
Schematic of the community water system used in this study. Initial distribution system evaluation (IDSE) monitoring sites from treatment stations A, C, and D were used to collect water samples for compliance reporting.

A total of eight residential sampling locations were included in the sampling plan based on their geographical connectivity with the treatment stations (Figure 1). Location 1 receives water from treatment station A; locations 2, 3, and 4 receive water from treatment station B; location 5 corresponds to D; location 6 corresponds to E; and locations 7 and 8 correspond to C. A total of 30 samples were collected during the sampling period (May–August), where 12 samples were from residential locations served by treatment station B, four by treatment station E, five by treatment station A, seven by treatment station C, and two by treatment station D. Except for treatment stations D and E, each treatment station had residential monitoring data collected from May through August. For treatment station D, the water samples were only collected in May and June, and for treatment station E, the water samples were collected only in June and July. Although we intended to collect a consistent number of tap-water samples from each treatment station during the study period, we failed to do so because of the unavailability of volunteers, noncompliance with the sample collection protocol, and damage to samples during shipment or transport. Thirty samples may seem small for this study, but it is substantially greater than what water suppliers collect in a given sampling period. For most public water suppliers, regulations require the collection of four samples in different locations once per quarter.

The water age in the distribution system may affect the quality of the finished drinking water [37]. Therefore, we also obtained data on water aging. The reported water aging for surface water systems varies from 1 to 4 days. This range is consistent with the American Water Works Association reported values of 3–7 days for a comparable water system that serves 800,000 people. [38] The Water Industry Database indicates an average water distribution system retention time of 1.3 days and a maximum retention time of 3.0 days based on a survey of more than 800 U.S. utilities [39].

### 2.2. Sampling Plan and Laboratory Analysis

The participants at each treatment station were randomly selected to participate in the study. They were provided training for sample collection, storage, and shipment. They received a sampling kit, tap water collection protocol, and a code to preserve anonymity. The sampling protocol included color coordinated diagrams with associated labels, making it easier for volunteers to understand and execute sampling accurately as per the laboratory standard procedure. On a given sampling day, we collected tap water samples from multiple participants. Sampling days occurred weekly or biweekly depending upon the availability of participants. All of the samples in the study were collected between 5 am and 8 am. The sampling kit included two vials, first for HAAs testing, and second for THMs testing. The sampling kit also included preservative hydrochloric acid (HCl) for THMs, and a proportionate number of ice packs for temporary storage. At lower pH, no THM formation is expected to occur in water [40]. Therefore, HCl was used as a preservative to minimize excess formation of THMs during storage. Samples were shipped on the same day for analysis to an EPA-certified laboratory for water testing. The samples were analyzed within 7 days of the collection date using the EPA recommended methods for THMs (# 524.2) and HAAs (# 552.2) [41,42].

### 2.3. Collection of Compliance Data

Both QA and LRAA data for THMs and HAAs were provided by the water supplier for the same year. Through treatment stations’ compliance with the Stage 2 DBP rule, they associated compliance sampling locations within the distribution system with treatment stations A, C, and D. However, we used the same compliance data as an indirect measure for treatment station B and E because they are also part of the same distribution network.

### 2.4. Data Analyses

All data analyses were conducted using R (v 3.1.2) (R Foundation for Statistical Computing, Vienna, Austria) and Microsoft Excel^®^ 2013 (Microsoft, Sacramento, CA, USA). We included two datasets in our analyses: (i) residential tap water monitoring data on total and individual THMs and HAAs, and (ii) compliance reported QA and annual LRAA data. We assessed the following: (a) the summary statistics of the measured levels of total THMs and HAAs, (b) comparison of the measured levels of total THMs and HAAs with the QA and LRAA data, and (c) short-term temporal and spatial variability in individual THMs and HAAs.

Summary statistics on the concentration distribution of total THMs and HAAs were calculated per treatment station for the 4-month sampling period. The temporal variability of total THMs and HAAs was represented by time-series graphs depicting monthly mean concentrations per treatment station and was plotted against QA and LRAA data. Similarly, we compared the spatial variability of THMs and HAAs in comparison with the LRAA data and calculated the significance. Individual THMs and HAAs were evaluated for temporal and spatial differences based on percent deviation in measured residential levels. A two-tailed Student’s *t*-test (α = 0.05) was conducted to determine statistical significance between any two treatment stations for total THMs and HAAs concentrations.

## 3. Results

### 3.1. Summary of the Residential Monitoring Data

The measured levels of total THMs and HAAs in residential tap water samples were used to summarize the mean concentrations for the entire monitoring period (May–August) by each treatment station for their use in analyzing the central tendency and variability in THMs and HAAs (Table 3).

The monitoring data shows a large range of concentrations across treatment stations (THMs Min = 16.19 µg/L, Max = 98.71 µg/L; HAAs Min = 11.17 µg/L, Max = 92.49 µg/L). Residential samples associated with treatment station D had the highest mean concentration of THMs (80.98 µg/L) and HAAs (62.83 µg/L), while samples from station E exhibited the lowest mean concentration of THMs (26.93 µg/L) and HAAs (16.69 µg/L). Each of the surface water locations showed significantly higher concentrations of THMs and HAAs than the groundwater location. The comparison of treatment stations shows that the location associated with station A displayed the highest variability for both THMs (SD = 22.09, CV = 0.57) and HAAs (SD = 26.67, CV = 0.32). Although, mean concentration levels for the sampling period rarely exceeded the EPA maximum concentration limit (MCL) for THMs (80 µg/L) and HAAs (60 µg/L), several individual water samples exceeded MCL. More than a quarter of the surface water samples, 27%, exceeded the MCL for THMs, with stations D and B showed the highest number of exceedances (>42%). The water samples coming from stations C and E never exceeded the MCL for THMs. All of the surface water locations showed a large range of HAAs concentrations (range: 19.88–92.49 µg/L) and 35% of the samples associated with surface water locations exceeded the HAAs MCL. The treatment station E had the lowest mean concentration of HAAs (16.69 µg/L) and never exceeded the HAAs MCL.

### 3.2. Temporal and Spatial Assessment of Total THMs and HAAs

**Figure 2:**
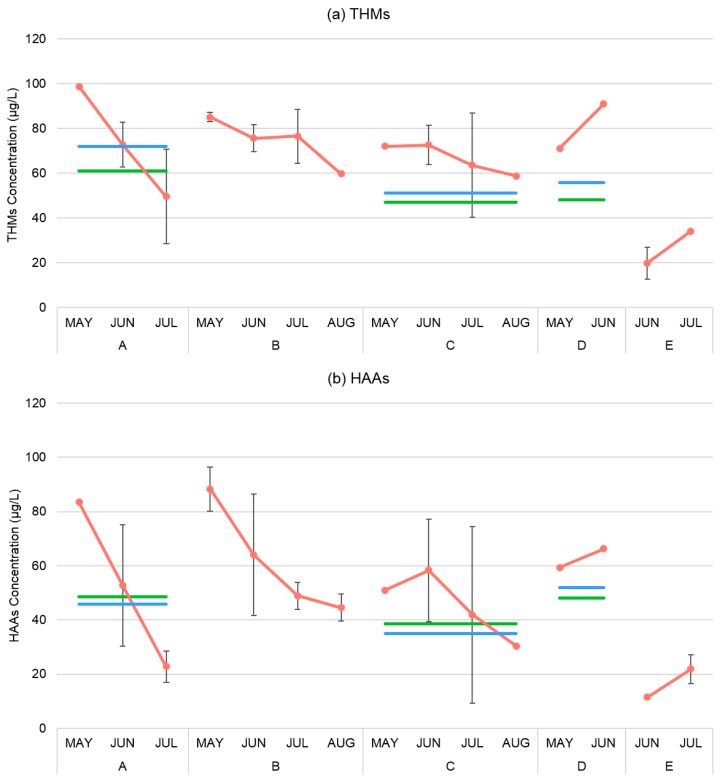
Temporal comparison of monitored (**a**) total THMs and (**b**) HAAs levels with LRAA compliance and QA data.

The temporal and spatial variability in the total THMs and HAAs levels were evaluated for the monitoring period. Residential tap water samples were analyzed to estimate monthly mean concentrations of total THMs and HAAs. For each treatment station, the corresponding monthly monitoring data from each location were pooled to obtain total THMs and HAAs. Mean monthly THMs concentrations ranged from 26.93 µg/L to 80.98 µg/L and mean HAAs concentrations ranged from 16.69 µg/L to 62.82 µg/L across all the treatment stations during the sampling period. Monthly mean concentration data (short-term) were compared with long-term LRAA and QA data calculated for treatment stations A, B, and D (Figure 2).

The green and blue lines represent the LRAA and QA reported data for systems A, C, and D, respectively; the error bars represent the 95% confidence interval of the mean of multiple samples (experimental error). Although the LRAA compliance sampling locations within the distribution system were associated with treatment stations A, C, and D, the same compliance data will be used as a proxy for treatment station B and E because they are also the part of the same water distribution system.

Treatment stations A, B, and C consistently have higher mean concentrations of THMs and HAAs in May followed by a gradual decline in June, July, and August. We only obtained mean concentrations for two months for systems D and E and therefore did not measure the fluctuations over time for these systems. The mean concentrations of A, C, and D were higher than the corresponding QA and LRAA compliance data at every measurement. We observed large differences in the monthly mean concentrations of THMs and HAAs across treatment stations A, B, and C, with a large coefficient of variations (CV; for THMs: CV range (0.33–0.57); for HAAs: CV range (0.14–0.32)).

**Figure 3:**
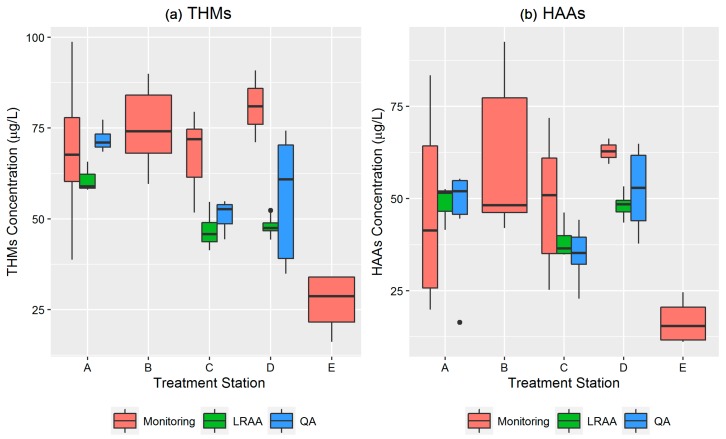
Spatial comparison of monitored (**a**) total THMs and (**b**) HAAs levels with LRAA compliance and QA data. The boxplots above utilize Tukey-style whiskers in that the upper whisker extends from the upper hinge (75th percentile) to the highest value that is within 1.5 × IQR (interquartile range) of the hinge and the lower whisker extends from the lower hinge (25th percentile) to the lowest value within 1.5 × IQR of the hinge. The black dots are outlier data points (outside the 1.5 × IQR range).

The residential monitoring data for the total THMs and HAAs for the entire sampling period was compared with the QA and LRAA data for each treatment station (Figure 3). With few exceptions, the monitoring data consistently showed higher concentrations than the QA and LRAA compliance data for each station. The comparison of monitoring data from all surface water stations (A, B, C, and D) with QA and LRAA showed significant differences in THMs and HAAs concentrations (*p* < 0.05). On the contrary, the monitoring data from groundwater station E consistently showed lower concentrations than the QA and LRAA compliance data. Also, these differences between monitored and compliance levels were statistically significant (*p* < 0.05).

### 3.3. Temporal and Spatial Assessment of Individual THMs and HAAs

Temporal assessment of individual THMs and HAAs was performed by calculating the monthly mean and standard deviation (SD) for each treatment station (Table 4). The percent deviation across all surface water treatment stations (A, B, C, and D) was calculated based on the highest reported SD for the ground water station (E). Ground water does not show high variability due to low contents of natural organic matter and inorganics (e.g., iron and manganese), and moderate temperature. Thus, we assumed that the highest reported variability (SD) for each chemical in the ground water station as the baseline variability to compare with the surface water stations. Overall, most chlorinated THMs and HAAs show higher temporal variability than brominated THMs and HAAs. Among all the regulated THMs, TCM is the major driver of variability in surface water (75%). For the regulated HAAs, both chlorinated and brominated HAAs show high temporal variability. However, TCAA (100%), and DCAA (87.5%) were the major drivers. For all surface water stations, we found high temporal fluctuations for individual TCM (0.8–16 µg/L), TCAA (1.1–11 µg/L), and DCAA (1.1–13.7 µg/L).

**Figure 4:**
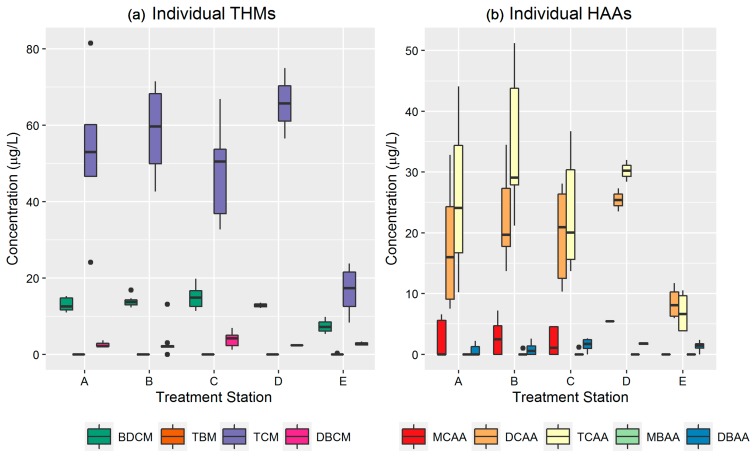
Spatial distribution of (**a**) individual THMs and (**b**) HAAs. BDCM = bromodichloromethane, TBM = bromoform, TCM = chloroform, and DBCM = dibromochloromethane. MCAA = monochloroacetic acid, DCAA = dichloroacetic acid, TCAA = trichloroacetic acid, MBAA = monobromoacetic acid, and DBAA = dibromoacetic acid. The boxplots above utilize Tukey-style whiskers in that the upper whisker extends from the upper hinge (75th percentile) to the highest value that is within 1.5 × IQR of the hinge and the lower whisker extends from the lower hinge (25th percentile) to the lowest value within 1.5 × IQR of the hinge. The black dots are outlier data points (outside the 1.5 × IQR range).

The spatial assessment of individual THMs and HAAs was performed by plotting the average concentrations of individual THMs and HAAs for the entire monitoring period for each treatment station (Figure 4). The assessment of monitoring data for individual DBPs across different treatment stations highlights that TCM, TCAA, and DCAA are the major drivers of spatial variability. Brominated species such as DBCM and BCAA show the least spatial variability (*p* < 0.05). Since, DBP compliance does not require the reporting of concentration levels of individual THMs and HAAs, we were not able to compare temporal and spatial differences between monitored levels of individual THMs and HAAs with LRAA and QA.

## 4. Discussion

The comparison of frequently measured residential mean concentrations of THMs and HAAs over the course of a summer period shows large deviations from the maximum allowable concentration limit (Table 2). The measured concentrations of total THMs and HAAs exceeded the MCL for several surface water systems. The deviation was significantly higher for THMs (CV = 0.57) than HAAs (CV = 0.32) for most surface water systems because of high natural organic contents and residual chlorine in the distribution system. On the contrary, HAAs show relatively low deviation in chloramine distribution systems. HAA concentrations decrease as they approach the farthest points in the distribution system due to microbial degradation [43]. Serodes et al. (2003) assessed the spatial variability of HAAs in two distribution systems using two different disinfection methods (chloramine and chlorine) [44]. In both systems, HAAs increased, followed by a gradual decrease, a phenomenon probably related to biodegradation. There is also evidence that iron pipes within a chlorinated distribution system can cause the abiotic reduction of HAAs. This could explain the lower but still positive deviation that this study found between frequently measured mean concentrations of HAAs and LRAA and QA data.

Temporal analysis of monthly mean concentrations of regulated THMs and HAAs measured at residential locations in this study suggest that May and June are high months of DBP formation in this system, which is consistent with other surface water systems with similar seasonal patterns of rainfall [14,43,44,45,46,47]. Treatment stations A, B, and D show high mean monthly concentrations in May (June in some cases, e.g., THMs in station D and HAAs in stations B and D). Spatial analysis of all surface water treatment stations show significantly higher concentrations for THMs and HAAs than the groundwater treatment station. This temporal and spatial variability was consistent with other studies conducted for different water systems, which have reported high temporal and spatial variability of THMs and HAAs in the summer and spring months [14,48]. High natural organic matter, water temperature [49], pH, and chloramine dose [50] may explain the high temporal and spatial variability in the surface water systems.

The comparison of monthly mean concentrations during the sampling period with corresponding QA and LRAA compliance data suggests that all surface water treatment stations in this study underestimate residents’ exposure to THMs and HAAs during some summer months (Figure 2). Conversely, the treatment system sourced from groundwater showed low variability and lower mean concentrations of THMs and HAAs because it requires a low chlorine dose and contains low dissolved organic matter, stable pH, and stable year-round temperatures (Table 1). Therefore, populations that rely on the groundwater supply are not exposed to high DBPs and the distribution system exhibits low spatial and temporal variability. Thus, the LRAA and QA compliance data overestimate the exposure for residents who depend on groundwater based public water supplies. In contrast, due to high temporal and spatial variability in surface water based treatment stations, frequent monitoring is essential in the summer months to capture high concentrations of THMs and HAAs. Assuming that the same compliance data derived from treatment stations A, C, and D can be used as an indirect measure for the population that relies on treatment stations B and E, it will underestimate the population exposures of THMs and HAAs for the sampling months for station B and overestimate them for station E. This may lead to a potential exposure misclassification issue in the populations that rely on these two systems.

The temporal and spatial analysis of individual THMs and HAAs is essential to understand which chemical is driving overall THM or HAA variability in the water distribution network. This may be helpful to improve the sampling strategy to capture individual DBPs with seasonally high variability. Major individual chemicals such as TCM, DCAA, and TCAA are the key drivers of temporal and spatial variability in this chloramine distribution system (Table 4 and Figure 4). A majority of other chloramine and chlorinated water treatment systems show a similar pattern of individual THM and HAA distribution. For example, the seasonal concentrations of individual THMs in a chlorinated surface water system in Istanbul, Turkey found that TCM is the main driver of THM variability in the system. Data collected from the summer months (June–August) indicate that TCM, DBCM, and BDCM levels are 1.2 to 1.8 times higher than in the fall (September–October) and spring (March–May). Summer months also show spatial variability in the range of 1.2 to 1.8 for both individual and total THMs [46]. This is consistent with our findings that show variability in the range of 1.3–1.6, with TCM being the largest contributor to THM concentrations, followed by BDCM, DBCM, and TBM. At three distinct geographical locations in the U.S., TCM shows the highest variability in the three water systems [51]. In Quebec City, Canada, individual TCM (SD ± 40), TCAA (SD ± 22), and DCAA (SD ± 16) show high temporal fluctuations (standard deviation ranges from 16 µg/L to 40 µg/L) in the distribution systems with levels that pose significant health concerns for small for gestational age [19].

However, these studies did not look at the temporal and spatial variability of individual HAAs to compare them with the monitoring data on individual HAAs. Most water supply providers are not required to submit individual DBP data along with their quarterly compliance reports. The absence of individual level DBP data further limits our understanding of individual DBP behavior in the distribution system. It also limits the usability of compliance data to assess the role of individual THMs and HAAs in causing adverse health effects. Several epidemiologic studies have shown an increased association between individual THMs and HAAs with adverse birth outcomes at the measured levels. For example, the consumption of tap water during pregnancy, containing different levels of BDCM, elevates the risks of neural tube defects (at >20 µg/L Risk Ratio (RR) = 2.5) [25], and spontaneous abortion (at >18 µg/L Odd Ratio (OR) = 2.0) [23] and (at >5 µg/L OR = 1.1–1.2) [16]. The concentrations of TCM > 36 µg/L [17], DCAA > 18 µg/L, and TCAA > 17.8 µg/L increased the risk of small for gestational age (OR = 0.95–1.14). Similarly, the consumption of drinking water contaminating DBAA (>5 µg/L) during weeks 33–40, and DCAA (>8 µg/L) during weeks 37–40 of pregnancy evaluated the risk of low birthweight and intrauterine growth restriction [22].

### 4.1. Limitations of the Study

Although we collected multiple tap water samples each week for several weeks, we were not able to recruit people from the extreme ends of the distribution system. We also lacked precise information about the distance between each sampling location and the corresponding treatment station. Most of our residential sampling locations were located within an 8-mile radius of the treatment stations; therefore, we were not able to establish intra-day spatial distribution of THMs and HAAs levels across different locations. However, we suspect higher THMs formation at locations furthest from the treatment station because of residual chlorine in the distribution system and relatively long contact time. Another limitation of our study is that the monitoring period was restricted to the late spring and summer months and thus it cannot be generalized to the relationship between compliance data and more-frequently gathered data in other months. Therefore, the findings from this study are only meaningful for the specified sampling period when THMs and HAAs peak in the drinking water. Also, this study was designed to compare frequently measured short-term levels with sparsely collected compliance data to evaluate the effectiveness of the compliance data for short-term exposure assessment applications for reproductive and developmental investigations. Thus, the generated monitoring data should not be used for exposure assessment applications.

### 4.2. Strengths of the Study

We can identify four strengths of our study that prior research has not offered. First, instead of sampling water from the distribution system, we collected tap water samples from residents. This type of sampling captures the excess formation of THMs and HAAs in the distribution system due to residual chlorine. Second, we collected water samples from both surface and groundwater based sampling locations. This allows us to compare short-term monitoring data with compliance data and captures differences in THMs and HAAs levels in surface and groundwater stations, which produce water with a wide difference in natural organic matter presence. Third, we collected repeated samples from each location in a relatively short time period. This helps to capture a range of concentration levels and average concentrations at each location, which minimizes bias in our measured levels. Fourth, this is the first study that compares frequently collected monitoring data directly with LRAA compliance and QA data for the same year. The implication of this work is significant as it provides the first direct comparison between long term (LRAA compliance and QA data) and short-term (few weeks) data on THMs and HAAs.

## 5. Conclusions

The findings from residential monitoring are in accordance with our initial hypothesis that QA and LRAA compliance data do not capture short-term high concentrations of THMs and HAAs. Despite having no known difference in natural organic matter, water ageing, pH, temperature, disinfection methods, or dose, all surface water based treatment stations showed high temporal and spatial variability for total and individual THMs and HAAs. Utilizing a relatively large number of collected samples in the short term (few weeks), we were able to capture the temporal and spatial variability that one sample per quarter, as current regulations require, would not capture in high DBP months. Although this evidence is suggestive, our results indicate that chronic LRAA and/or QA data are not a good measure for short-term acute exposure. More frequent monitoring, especially in surface water systems, could help to capture the short-term temporal and spatial variability in individual and total THMs and HAAs, and to an extent, minimize exposure misclassification. However, further investigation is warranted to determine if other public water systems show similar patterns so that compliance can be designed to capture reliable short-term exposure for developmental health outcomes research. Most countries have similar compliance for THMs and HAAs, and often lack short-term data on regulated THMs and HAAs. Hence, the findings from this study can also be applied to other geographical water supply systems to evaluate the usefulness of compliance data to estimate representative short-term exposure of regulated DBPs. Also, further research on the chemical nature of surface and ground water born natural organic matter will help to understand the deep differences in DBP formation and their toxicities for research on their health effects.

## Figures and Tables

**Table 1:** Summary of key birth cohort studies on THMs and HAAs with inconclusive findings.

Study	DBPs Type	Birth Outcomes Examined	Findings	Study Location
Dodds and King, 2001 [25]	THMs, TCM, BDCM	Neural tube defects, cardiovascular defects, cleft defects, chromosomal abnormalities	No statistically significant association was found with any of the congenital anomalies	Nova Scotia, Canada
Cedergren, 2002 [15]	THMs	Cardiac defects	Statistically significant association	Sweden
Waller et al., 1998 [23]	THMs	Spontaneous abortion	Only high BDCM exposure (>18 µg/L) was associated with spontaneous abortion	USA
Savitz et al., 1995, 2006 [26,27]	THMs	Miscarriage, preterm birth (PTB), low birth weight (LBW)	No statistically significant association	North Carolina, USA
Wright et al., 2004 [16]	THMs and HAAs	Mean birth weight (MBW), mean gestational age, small for gestational age (SGA), PTB	Elevated mutagenic activity was associated with SGA (odd ratio = 1.25; 95% confidence interval (CI), 1.04 to 1.51) and MBW (−27 g; 95% CI)	Massachusetts, USA
Hoffman et al., 2008 [21]	THMs and HAAs	SGA	Analysis did not show a consistent association	USA
Patelarou et al., 2011 [28]	THMs	LBW, SGA, PTB	No significant association was found	Crete
Grazuleviciene et al., 2011 [29]	THMs	Congenital anomalies	No significant association was found	Lithuania
Rivera-Nunez and Wright, 2013 [17]	THMs, HAAs, brominated THMs (BrTHMs)	Mean birth weight, SGA, PTB	Statistical association was found between BrTHMs and mean birth weight	Massachusetts, USA
Costet et al., 2012 [18]	THMs and TCAA	Fetal growth restriction (FGR), PTB	Higher uptake BrTHMs was associated with FGR	France
Porter et al., 2005 [30]	THMs and HAAs	Intrauterine growth retardation (IUGR)	No statistically significant association	Maryland, USA
Levallois et al., 2012 [19]	THMs and HAAs	SGA	Increased risk was observed	Quebec, Canada
Horton et al., 2011 [24]	THMs and HAAs	SGA and PTB	No association was observed	North Carolina, USA
Luben et al., 2008 [31]	THMs and HAA	Hypospadias	No association was found	Arkansas, USA
Hinckley et al., 2005 [22]	THMs and HAAs	LBW and IUGR	Dibromoacetic acid and dichloroacetic acid show association with LBW	Colorado, USA
Hoffman et al., 2007, 2008 [20,21]	THMs and HAAs	SGA	Only THMs were associated SGA	USA

**Table 2:** Physicochemical properties of raw and finished drinking water.

Treatment Station	Water Type	pH		% UV Transmittance		TOC mg/L		Temperature Degree F		Chlorine mg/L	
		Raw	Finished	Raw	Finished	Raw	Finished	Raw	Finished	Raw	Finished
A	SW	7.94	7.37	75.76	91.49	4.25	2.46	74.64	74.94	7.0	1.9
B	SW	8.07	7.46	81.12	90.57	3.48	2.41	71.43	75.49	5.5	1.5
C	SW	8.11	7.55	69.03	90.53	3.63	2.59	74.92	75.03	5.0	2.2
D	SW	8.22	7.63	77.42	90.84	3.63	2.10	72.78	68.59	5.6	2.0
E	GW	7.34	7.66	N/A		N/A		58.32	N/A	1.6	1.5

SW and GW represent surface water and ground water sources, respectively. Total organic carbon (TOC) and temperature data were calculated using the seasonal average (May–August) data from 2011–2015. pH and % UV (Ultra-violet) Transmittance data were calculated using the seasonal average (May–August) data from 2011–2014 and 2014–2015, respectively. In the surface water treatment stations, the chlorine in raw water represents total residual chlorine. It was estimated based on the chlorine demand of the water, and added at multiple locations in the disinfection process. The chlorine in finished drinking water represents the free chlorine, preventing the growth of algae, aiding in the coagulation of organic substances and reducing odor. For groundwater, the chlorine demand does not change significantly from raw to finished water, possibly because of low levels of natural organic matter, iron, and manganese.

**Table 3:** Summary statistics of the residential monitoring data for total THMs and HAAs.

Treatment Station	Monitoring Location	Number of Samples	Mean	Min	Max	Median	GM	SD	CV	95% CI	Number of Samples >MCL	% Samples >MCL
**Total THMs (µg/L)**												
A	1	5	68.65	38.77	98.71	67.63	65.60	22.09	0.57	42.65–100.90	1	20
B	2,3,4	12	74.86	59.60	89.90	74.08	74.14	10.66	0.33	67.59–81.33	5	42
C	7,8	7	67.93	51.74	79.45	71.95	67.26	10.02	0.36	58.28–77.62	0	0
D	5	2	80.98	71.10	90.86	80.98	80.38	13.97	0.22	16.92–381.72	1	50
E	6	4	26.93	16.19	34.07	28.74	26.76	8.73	0.38	14.62–45.38	0	0
**Total HAAs (µg/L)**												
A	1	5	46.95	19.88	83.46	41.38	40.85	26.67	0.32	19.36–86.20	2	40
B	2,3,4	12	59.76	42.00	92.49	48.21	57.05	19.86	0.14	46.84–69.49	4	33
C	7,8	7	48.60	25.29	71.86	50.93	45.68	17.42	0.15	31.79–65.64	2	29
D	5	2	62.82	71.10	90.86	62.82	62.72	13.97	0.17	31.41–125.23	1	50
E	6	4	16.69	11.17	24.63	15.47	15.79	6.42	0.32	8.58–29.05	0	0

GM = Geometric Mean, SD = Standard Deviation; CV = Coefficient of Variation; CI = Confidence Interval.

**Table 4:** Temporal variability of individual THMs and HAAs for all surface water stations.

Chemical	A				B				C				D				E				% Deviation
	May	Jun	Jul	Aug	May	Jun	Jul	Aug	May	Jun	Jul	Aug	May	Jun	Jul	Aug	May	Jun	Jul	Aug	
TCM	81.5	56.6 (5.1)	24.1 (15.9)	NA	70 (2.1)	60.3 (6.0)	57.6 (9.9)	43.2 (0.8)	NA	58.6 (8.5)	41.9 (12.9)	33.6	56.5	75.0	NA	NA	NA	11.6 (4.0)	22.3 (2.1)	NA	75.0
BDCM	16.30	13.6 (1.6)	11 (0.5)	NA	14.6 (0.7)	13.2 (0.8)	14.0 (1.9)	14.4 (0.4)	NA	12.1 (0.6)	17 (4.0)	18.3	12.1	13.6 (1.6)	NA	NA	NA	5.8 (0.7)	8.9 (1.3)	NA	37.5
DBCM	1.91	2.5 (5.1)	2.9 (1.3)	NA	0.9 (1.3)	2.0 (0.5)	5.0 (5.4)	2.1 (0.1)	NA	1.8 (0.6)	4.8 (0.2)	6.9	2.5	2.3	NA	NA	NA	2.7 (0.26)	2.8 (0.8)	NA	37.5
TBM	<LOD	<LOD	<LOD	<LOD	<LOD	<LOD	<LOD	<LOD	<LOD	<LOD	<LOD	<LOD	<LOD	<LOD	<LOD	<LOD	<LOD	<LOD	<LOD	<LOD	NA
MCAA	6.56	2.8 (3.9)	NA	NA	6.9 (0.4)	3.9 (1.3)	0.5 (1.1)	<LOD	NA	3.7 (1.4)	<LOD	<LOD	5.5	5.4	NA	NA	NA	<LOD	<LOD	NA	NA
DCAA	32.80	20.1 (5.9)	8.2 (1.1)	NA	32.4 (3.0)	23.2 (7.0)	19.1 (2.5)	16.9 (4.5)	22.2	25.2 (4.9)	18.0 (11.0)	12.7	23.5	27.3	NA	NA	NA	6.2 (0.2)	10.8 (1.3)	NA	87.5
TCAA	44.10	29.2 (7.3)	13.5 (4.6)	NA	46.5 (1.1)	36.0 (13.7)	28.9 (1.5)	27 (1.9)	22.1	28.6 (10)	22.0 (11.7)	15.3	28.4	32	NA	NA	NA	3.9 (0.1)	10.0 (0.8)	NA	100.0
MBAA	<LOD	<LOD	<LOD	NA	0.5 (0.7)	<LOD	0.3 (0.5)	<LOD	<LOD	0.4(0.7)	<LOD	<LOD	<LOD	<LOD	NA	NA	NA	<LOD	<LOD	NA	NA
DBAA	<LOD	0.6 (0.9)	1.1 (1.5)	NA	2.1 (0.7)	0.6 (0.7)	0.4 (0.7)	0.7 (1.0)	2.1	0.5 (0.8)	1.9 (0.8)	2.4	2	1.5	NA	NA	NA	1.5 (0.1)	1.2 (1.7)	NA	0.0

where, BDCM = bromodichloromethane, TBM = bromoform, TCM = chloroform, and DBCM = dibromochloromethane. MCAA = monochloroacetic acid, DCAA = dichloroacetic acid, TCAA = trichloroacetic acid, MBAA = monobromoacetic acid, and DBAA = dibromoacetic acid. A, B, C, and D, are surface water treatment stations (SW), and E is ground water (GW) treatment station. % Deviation represents the deviation between concentration levels of each chemical across different SW stations. The following equation was used to calculate percent deviation: % Deviation = (number of SW data points with standard deviation (SD) greater than the highest reported SD in GW/(total number of SW data points with SD) × 100. All the SDs for SW stations are shown in parentheses. NA represents no data available. <LOD represents reported data below the limit of detection. The highest SDs (µg/L) for each chemical in GW (station E) are as follows: TCM = 4.0, BDCM = 1.3, DBCM = 0.8, DCAA = 1.3, TCAA = 0.8, and DBAA = 1.7. For the remaining chemicals in GW, the levels were below <LOD.

## Abbreviations

LRAA	Locational running annual average
QA	Quarterly running average
DBPs	Disinfectant Byproducts
THMs	Trihalomethanes
TOC	Total organic carbon
HAAs	Haloacetic acids
MCL	Maximum concentration limit
IDSE	Initial distribution system evaluation
BDCM	Bromodichloromethane
TBM	Bromoform
TCM	Chloroform
DBCM	Dibromochloromethane
MCAA	Monochloroacetic acid
DCAA	Dichloroacetic acid
TCAA	Trichloroacetic acid
MBAA	Monobromoacetic acid
DBAA	Dibromoacetic acid
HCl	Hydrochloric acid
SGA	Small for gestational age
PTB	Preterm birth
FGR	Fetal growth restriction
LBW	Low birthweight
IUGR	Intrauterine growth retardation
GM	Geometric Mean
SD	Standard deviation
CV	Coefficient of variation
CI	Confidence interval
